# Exploring bison producers' access to veterinary services in Ontario, Canada

**DOI:** 10.3389/fvets.2024.1448216

**Published:** 2024-12-11

**Authors:** Natassia H. Lambrou, Charlotte B. Winder, Kelsey L. Spence

**Affiliations:** Department of Population Medicine, Ontario Veterinary College, University of Guelph, Guelph, ON, Canada

**Keywords:** epidemiology, bovine, accessibility to care, veterinary medicine, producer perspectives, buffalo

## Abstract

**Introduction:**

Access to veterinary services is integral for animals of all species. These services play a crucial role in maintaining their health and welfare and maintaining a healthy, safe, and sustainable food system. Research has consistently shown that rural communities face challenges accessing veterinary services, with livestock producers outlining several barriers including cost, inadequate infrastructure, and delays in receiving treatments. Research on bison producers' access to veterinary services is limited, prompting our investigation to address this gap in knowledge. This qualitative study aimed to describe Ontario bison producers' current access to veterinary services and identify how any barriers, as perceived by producers, might impact their herd health and management practices.

**Methods:**

Ontario bison producers were invited to participate in virtual focus groups to share their perspectives on their access to veterinary services. Audio from the focus groups was recorded, transcribed verbatim, and analyzed using reflexive thematic analysis.

**Results:**

Despite all participants indicating they had access to veterinary services, they also encountered obstacles and expressed concerns accessing and utilizing these services. Two overarching themes were identified: producers were concerned about the future stability and costs associated with bison farming, and they had a desire to improve bison-specific knowledge among veterinarians servicing their farms. Producers suggested several strategies to address these concerns, including improving collaborations with veterinary organizations, like the College of Veterinarians of Ontario, to increase veterinarians' exposure to bison, building stronger relationships between producers and their veterinarians, monetary incentives for established food animal veterinarians, and providing financial support to prospective food animal veterinarians.

**Discussion:**

The findings of this study demonstrate that although bison producers in our sample had access to veterinarians, they may not fully utilize the services or find their access entirely beneficial. Future research into the veterinarian perspective would allow for greater insights into these barriers, adding additional value and contributing to a more wholistic understanding of the topic.

## 1 Introduction

Bison, a large species of bovine, are the largest terrestrial animals in North America (1). Wild bison can be found in grasslands and savannahs, and through their grazing and wallowing behaviors play an important role in many ecological processes (2, 3). While farmed bison are a niche species, they are becoming more popular in Canada. From 2016 to 2021, the farmed bison population in Canada grew by about 25% (4). In 2022, it was reported there was ~150,000 head of farmed bison across 980 farms in Canada (5). Compared to other species of bovine, bison are more resilient to colder temperatures due to physiological adaptations such as thicker coats and reduced metabolism during winter months (6, 7). Bison require less housing due to these adaptations and may appeal to some producers as an alternative meat production animal (3). The majority of a bison's nutritional requirements can be met through grazing perineal grasses that are typically grown in agricultural regions of Canada, which reduces the input costs of producing or purchasing other feedstuffs (2). As bison production continues to emerge across Canada, it is essential to ensure that producers have access to necessary veterinary services to maintain bison health and welfare (5).

Veterinary services are essential throughout all stages of animal production to aid in maintaining optimal livestock and herd health (8–11). Veterinarians can assist producers in the management and prevention of diseases, including those that may be zoonotic (8). Food animal veterinarians, in particular, play an important role in both the veterinary and public health sectors and can advise producers on herd health management strategies and biosecurity protocols (9, 10, 12). Although herd health management strategies have been proven to improve both animal productivity and welfare, some food animal producers may primarily utilize veterinarians for emergencies, which is an approach often referred to as “fire-engine” medicine (13–17). Previous research in other bovine species has found that veterinary involvement on farms can result in improved herd health and welfare, higher productivity, improved fertility, and early problem detection (18, 19). Bison share many similarities with other farmed bovine species, including dairy and beef cattle, but there are differences in both restraint and medical management of bison due to their physiology and lifestyle (20). These differences may make it difficult for producers to locate a veterinarian with special interest and expertise in working with and treating bison (21).

Research among other food animal producers such as dairy, beef, veal, poultry, sheep, and swine have identified several consequences of reduced access to veterinary services, including negative impacts to herd health and welfare, inability of producers to create and maintain a veterinarian-client-patient relationship, and limited options for alternate medical consultations or more cost-effective care (11, 22). A 2022 survey of food animal producers in rural Ontario reported that a lack of veterinarians and limited to no access to veterinary care were major barriers among respondents (11). Similarly, a study of livestock producers' views on accessing food animal veterinary services reported barriers including timeliness of services, travel expectations to veterinary clinics, and overall cost of visits (23). While previous research provides insights into barriers faced by other livestock types, little is known about bison producers specifically. Proposed solutions for addressing barriers to accessing veterinary services have included monetary incentives for rural-based veterinarians, scholarships for veterinary students and increasing food animal veterinary technicians (12, 23). However, little is known about whether bison producers experience similar barriers as other livestock producers, and if so, whether these proposed solutions would meet their needs.

The objectives of this study were to describe Ontario bison producers' current access to veterinary services, identify any barriers that producers may face accessing veterinary services, and explore how any barriers might impact producers' herd health and management practices.

## 2 Methods

This was a qualitative focus group-based study where bison producers were invited to share their perspectives on their access to veterinary services in Ontario. The study was reviewed and approved by the University of Guelph Research Ethics Board (REB#23-05-002). The methods and results of this study were reported based on the consolidated criteria for reporting qualitative research (COREQ) (24).

### 2.1 Theoretical framework

This study was conducted from a critical realist paradigm, which acknowledges that established theories alone cannot shape our understanding of knowledge, and instead should be integrated with empirical evidence to further our understanding (25). This paradigm aligned with our approach to account for producers' perspectives and any potential influences that their experiences may have on these perspectives. Strong retroductive reasoning was used in conjunction with this framework, allowing us to explore and try to understand the underlying rationale behind their perspectives. Using this technique, we were able to further examine potential underlying explanations that may affect producers' access to veterinary services.

### 2.2 Participant selection

Convenience sampling was used to select participants for this study. According to the Ontario Bison Association, there were 35 bison producers located in Ontario at the time of the study (26). Producers' email addresses were provided by the Ontario Bison Association and were used to send invitations to participate in the study. Producers were contacted via email several times over a two-week period. Due to the small source population size, sampling ceased when there were no more willing participants available. As such, no interim analyses to evaluate data saturation were performed. To participate in the study, participants had to be at least 18 years old, be able to speak and understand English, and be a bison producer located in Ontario, Canada.

### 2.3 Data collection

Focus group sessions with participating bison producers were conducted between September and October 2023. The research team was experienced in conducting qualitative research focused on livestock producers and their animals. The team included coauthors CBW (an epidemiologist and food animal veterinarian) and KLS (an epidemiologist specializing in infectious disease and biosecurity). The focus groups were conducted and facilitated by the first author (NHL), who was a Master of Science (MSc) student at the time of the study and had received training in qualitative study methodology. Although informed by her master's training in epidemiology and previous work experience conducting focus groups, NHL was unfamiliar with both bison farming and producer perspectives. This information was disclosed to participants prior to data collection.

A semi-structured question guide was developed by the research team and was intended to generate discussion about producers' current access to veterinary services, perceived barriers to accessing veterinary services, and how this might influence their herd health management strategies. A copy of the focus group guide is provided in Supplementary Data 1. Having access to veterinary services was defined by the research team as, “Being able to access a veterinarian (and their services) for participating herds should they need it.” Producers were given this definition during the informed consent process and reminded about the study's purpose prior to commencing the focus group. The question guide was not pilot tested with bison producers due to concerns about the limited number of available participants; however, the comprehension and flow of the questions was reviewed by the research team and a simulated pilot with graduate students was done prior to launch.

Once producers agreed to participate in the study, they were organized into focus groups based on their experience and time spent in the industry, with the objective of having a diverse representation of both characteristics in each group to ensure a balanced discussion (27). Prior to the start of each focus group, producers were reminded of the objective of the study, how “access to veterinary services” was being defined in this study, given a brief synopsis of the previously distributed consent document, and asked to verbally reaffirm their consent to participate. The focus groups were conducted using the online platform Zoom version 5.16.2 (Zoom Video Communications Inc, California, US) allowing participants to join through internet or dial-in using their phones. There were no other producers or observers present other than the participants and NHL (the facilitator). Audio recordings of the focus groups were made, and NHL kept notes regarding each session to aid with data interpretation. The audio recordings were uploaded to Otter.ai (Otter.ai, Inc., California, US) and were automatically transcribed verbatim using the software. The transcripts were reviewed against the original audio for accuracy by NHL and a research assistant. The transcripts were not returned to participants for member checking due to their limited availability.

### 2.4 Data analysis

Transcripts were uploaded to the software program NVIVO version 14.23.2 (Lumivero, Colorado, US) to facilitate data analysis. Reflexive thematic analysis was used, allowing for an inductive approach to identify themes within the data (28). All primary analyses were conducted by NHL. Prior to the coding process, transcripts were re-read and initial thoughts or ideas were noted while reading. Initial codes were generated by coding the data line by line. The initial codebook included a label, a descriptive label (when necessary), and quotes associated with the code. After coding, the relationship between codes was explored through the creation of a thematic map, which was used for theme development. Four candidate themes with supporting quotes and annotations were established; these themes were then reviewed and modified to better reflect the data. We noted overlap between some of the candidate themes, so they were further refined into two main themes. Finally, the research team reviewed the content within each theme and identified the appropriate titles to best reflect the data. Defining and renaming the themes took multiple rounds of revision until the final wording was confirmed. In addition to having discussions amongst the research team about potential assumptions and biases, a detailed log of theme generation rationale was kept. Reflexivity was practiced through the development of the methods, throughout the analysis and the manuscript generation.

## 3 Results

After recruitment concluded, there were seven producers who met the inclusion criteria and agreed to participate in the study. The final sample represented 20% (7/35) of the available population of Ontario bison producers. Participating producers had between 5 and 31 years of experience in the industry, and their herd sizes ranged from 8 to 85 head. All participants were male, and all had farms located across four pre-defined regions of Ontario (two in the west region, two in the central west region, one in the north region, and two in the east region) (29). Through the focus group discussions, it became clear that each participant had access to veterinary services in their region. However, they expressed concerns regarding access to bison-specific care, as reported in the two themes identified from the interview data.

### 3.1 Theme 1: concerns over future stability and costs associated with veterinary services

Based on their experiences in the industry, participants perceived that fewer new large animal veterinarians were entering the field compared to previous years. They believed this was due to small animal medicine being more lucrative and attractive to new veterinarians because of better hours and pay. Multiple participants echoed, and agreed with, a statement made by a producer on this perception:

“People going into large animal practice, I think they're becoming fewer”—Producer 1, Focus Group 1

Participants mentioned that the absence of new large animal veterinarians may cause issues regarding their access to veterinary services in the future. They indicated that this would need to be addressed to sustain the demand not only for the bison industry but for other livestock sectors. However, participants also acknowledged that their perceptions were shaped by their current access to veterinary services. Because of potential differences in access, they recognized that their concerns for their own herd may not reflect their concerns for other herds or producers. For example, one participant noted that while he had what he perceived to be good access to veterinary services, he was concerned that the industry may face issues with recruiting and retaining large animal veterinarians in the future:

“I'm lucky because the people are doing large animal practice at the vet I use, the young people. So, I'm fortunate that there's actually three different people there that are involved with large animal practice. I'm lucky. So, I think going forward that can become more of a problem, you know, with finding veterinarians that want to do large practice, because it's the path of least resistance. Some [think], ‘I can make enough money and I can do small animals, cats and dogs and things like that. And hamsters. Why would I go out and deal with an ornery 2000-pound animal?”'—Producer 1, Focus Group 1

As with any industry, producers must be able to sustain their production costs while still generating enough money to run their operation. When discussing post-mortem costs, one participant noted that the veterinary costs, paired with the loss of the cow, was unsustainable. Multiple participants mentioned the association between increasing costs and their willingness and ability to pursue veterinary services:

“I only [brought out a veterinarian] because I made it work. And the expenses weren't that high. But starting last year, the fees [associated with veterinary visits] have really gone up”—Producer 3, Focus Group 1

Several participants stated they would first contact another producer before calling a veterinarian if they had issues with their bison. Sometimes, calling another producer instead of, or before, a veterinarian stemmed from a perceived lack of effective treatment for the money spent on the service, as stated by one participant:

“No, if I have issues, I'd call up [bison producers] that I know, that I trust. Because vets are overpriced.”—Producer 7, Focus Group 2

However, another participant believed that the veterinarians' time was worthy of the increasing costs associated with farm visits:

“The call out fees [for vets] have definitely gone up. But it's like everything else, their time is worth money as well.”—Producer 3, Focus Group 1

Participants expressed that having a veterinarian who was familiar with bison, was able to correctly diagnose an ailment, and provide an effective treatment plan would be worth the increased cost of services. Multiple participants believed there was monetary value in having a veterinarian who was familiar with bison treat their herd:

“…But starting last year, the fees have really gone up. So one's going to have to really think twice whether or not, or how often they use a vet and what they use them for, because of the increasing costs. […] Our revenues haven't really been keeping up with our costs. I know for us, we haven't raised our meat prices in three years where we probably need to, but the farm income isn't matching the farm costs.”—Producer 7, Focus Group 2

### 3.2 Theme 2: desire to improve bison-specific knowledge among veterinarians servicing their farms

Most participants stated that the lack of bison-specific knowledge among the veterinarians that serviced their farms was the biggest barrier to accessing effective veterinary care for their herds. Participants said they would like for their veterinarian to become more familiar with bison in terms of species-specific handling, concerns, and ailments. Some participants stated that while they had a positive relationship with their veterinarian, and that their veterinarian was willing to treat their bison, they did not have substantial bison-specific knowledge:

“I would say our biggest barrier is lack of knowledge for the vets. And it's not lack of willingness from them. It's just general lack of knowledge.”—Producer 4, Focus Group 2

Participants described how their veterinarians typically used experience and knowledge of other bovine species, like beef and dairy cows, when treating bison. They speculated this may be due to the lack of specific knowledge or experience with bison among the veterinarians. A few participants had negative experiences with veterinarians, with one stating they felt most veterinarians did not know the differences in handling techniques between bison and beef or dairy cows. This lack of knowledge was perceived to create a negative experience for the bison, producers, and veterinarians. One participant described how his current veterinary team used a trial-and-error approach when treating bison, often relying on other cattle experience to diagnose ailments that may not be effective on bison:

“So yes, them coming and not having the knowledge and just kind of throwing a dart at a board and kind of guessing as to what they think it might be that relates to maybe cattle, but [might not] work for bison”—Producer 6, Focus Group 2

Because of the perceived lack of bison-specific knowledge among veterinarians, participants often discussed the health and issues with their herd with other producers before consulting a veterinarian. One participant mentioned they had a negative experience with a veterinarian a surrounding a misdiagnosis, which made them more likely to contact another producer regarding a health issue as opposed to a veterinarian. Participants felt that by contacting other producers first before contacting a veterinarian, they would be able to access someone with direct lived experience with bison:

“I went to producers first because our vet here, [he's] a super intelligent vet but only dealt with cattle and small animals. […] And he's a bible of knowledge, it's just, he's more of a cattle vet than he is a bison vet and I'm kind of like [the other participant], you know, [I'll] call the senior guys in Ontario Bison Association. Or we got animals out of Saskatoon [from another producer]. He's a brilliant guy. He's got 600 or 700 head out [west]. He's like, he's 75 years old, and he's had animals for 30 years and seen everything. He's seen pretty much everything that a bison producer could see in those 30 something years.”—Producer 5, Focus Group 2

A few participants noted they tend to their bison's health needs on their own, without seeking medical advice or a medical visit from a veterinarian. Some participants stated that they would not seek veterinary care due to perceived lack of bison-related knowledge and others due to a perceived lack of trust due to limited bison knowledge. Another participant used the general nature of bison to rationalize not seeking veterinary services often and his preference for a hands-off approach:

“The one vet, he's a very busy guy, and he doesn't know bison as well as I'd like him to. So really we do everything ourselves”—Producer 7, Focus Group 2

Some participants saw the value in involving a veterinarian with bison-specific knowledge to help with the treatment of their bison, since they acknowledged that this was beneficial to the health of their bison:

“They have bison specialists up there. So once he got involved, it was ‘yeah, we know exactly what it is, and here's what you have to do.' So that was definitely a huge help after that.”—Producer 6, Focus Group 2

Other producers noted that while they had timely access to veterinary services, they did not have access to veterinarians with bison-specific knowledge. Some participants noted they would contact a producer over or before a veterinarian based on both convenience and accessibility, since they would have to wait longer for a veterinarian with bison-specific knowledge:

“I'd probably call [another producer], he's close, he can come over and take a look. And then while I'm waiting for him, I'd probably call the vet just on backup.”—Producer 7, Focus Group 2

Some participants described how contacting veterinarians remotely improved their ability to access appropriate care. One participant described contacting a veterinarian from the United States (US) who had bison-specific knowledge and was able to assist remotely when the participant was unable to find local help. Another participant described using his local veterinarian in a telemedicine capacity, which worked well for his specific needs and allowed for timely access to veterinary services:

“Our vet's also super busy. So good thing for phones and data transfer. Like, we'll send a video like, ‘Hey, what do you think of this animal?' Send a video of it. You know, ‘this one's limping. What do you think of that?' Or, you know, send a couple pictures. And he'll be like, ‘yeah, do this, this and this”'—Producer 5, Focus Group 2

To improve bison-specific knowledge among their veterinarians, some participants described providing them with educational materials. Several participants suggested that collaboration with local bison producers would also help increase bison-specific knowledge among both established and incoming veterinarians. This was due to the perceived value in this collaboration, with multiple participants offering to have veterinarians and veterinary students come out to tour their farms and learn from them:

“[University of Guelph] has a program down there for veterinarians … And if they need to go see bison farms in the area, I'm pretty sure all they have to do is phone a few and find and ask if it's okay to if they do a field trip out there. You know, as long as they follow all the biosecurity measures that that farm has in place, then there should be no issue.”—Producer 7, Focus Group 2

## 4 Discussion

This exploratory study provides insight into Ontario bison producers' current perceptions of access to veterinary services, alongside several barriers to receiving appropriate veterinary care. Our findings highlighted several concerns by producers, including the perceived lack of new large animal veterinarians entering the field, the costs associated with veterinary services, the effectiveness of available care, and limitations to accessing veterinarians with species-specific knowledge.

Numerous participants described how their choice to not contact a veterinarian when experiencing herd health concerns was due to the cost associated with these visits. Some participants also felt that the quality or effectiveness of services being received did not align with their expectations. Cost is a commonly cited concern among other livestock producers. For example, a study of various US small-scale (gross annual agricultural sales between $10,000 and $499,999 USD) livestock producers, including dairy, beef, swine, sheep, goat and poultry, found that over 12% reported not pursuing veterinary care due to cost (30). A survey of Tennessee livestock producers, including beef, dairy, poultry, swine and horses, revealed that 18.8% didn't seek veterinary services as it was too expensive, while 38.6% found the services too expensive compared to the animal's value (23). These perceived increasing costs have also been noted by the American Veterinary Medical Association (AVMA) in companion animal practices, where in addition to the rising costs of companion animal veterinary services, there is a decline in the proportion of pets visiting a veterinarian for annual check-ups, driving an increase in the cost per visit when they do seek veterinary care (31). A 2015 report, looking at Canadian census data from 2006 and 2011, compared the cost of veterinary services to farmers to other farm operation costs such as: herbicides, insecticides, fungicides, feed/supplements, hay, livestock or poultry purchases, fuel expenses, utilities and miscellaneous (32). When compared to these, veterinary services accounted for 1.85% and 1.63% of total operating costs among Canadian producers in 2006 and 2011 respectively (32). While veterinary services costs seemingly make up a low percentage of overall farm costs, they are still an added expense to the producer and may contribute to their decision to pursue these services.

When the participants used veterinary services, many referenced only seeking assistance for emergency cases, called “fire-engine” medicine, rather than preventative medicine. Herd health management programs often incorporate elements of preventative medicine including vaccination protocols, pregnancy checks and parasite prevention programs (13, 14, 17, 33, 34). These strategies can be a cost-effective way to mitigate disease risk among ruminant herds like dairy, beef, sheep, goat, and African buffalo (24–28), suggesting they may have similar benefits among bison herds. The evolution of livestock veterinary medicine away from ‘fire-engine' medicine to implementing a ‘herd health' approach has been ongoing for decades, an evolution that can also been seen in veterinary education and curricula (15, 16, 35, 36). Despite this progress, veterinarians still face challenges with the implementation of farm-level preventative approaches. A scoping review conducted in 2023 suggested that one of the main barriers to adopting herd health practices among North American dairy and beef producers was the cost to producers (37). In a 2019 study examining barriers to implementing herd health management changes for Johne's disease control among Ontario dairy producers and veterinarians, the veterinarians noted cost as a prominent concern among producers (38). Another contributing factor to the implementation of herd health strategies is a strong veterinarian-producer relationship, where the veterinarian understands the producer's objectives and future goals for their herd (39–42). Studies have found farmers with greater knowledge are better equipped to solve problems, more autonomous in their decision-making, and have higher rates of cooperation and compliance with veterinarian recommendations (39, 43, 44). A greater understanding of herd health practices among bison producers may result in a higher likelihood of them prioritizing preventative health measures over emergency veterinary visits. The implementation of herd health strategies shows a positive return on investment (ROI) in dairy farms in California and across Europe, as well as swine farms across Europe (45–47). Providing bison producers with a preliminary cost-benefit estimate of preventative medicine, emphasizing the benefits, such as reduced disease risk and maximizing ROI, may help drive interest in the approach and develop stronger veterinarian-client-patient relationships (42). This is an area that warrants further exploration to determine the definitive impact of ROI for bison farmers.

Previous studies suggest that the majority of practicing veterinarians in both the US and western Canada are companion animal focused, with food animal veterinarians making up only ~10% of western Canadian veterinarians and ~4% of US veterinarians (48–51). The Canadian Veterinary Medical Association conducted a workforce study in 2020, which found that every Canadian province (excluding Prince Edward Island) indicated a need for food animal veterinarians (52). The report noted a trend where recent veterinary school graduates are more likely to pursue companion animal medicine rather than food animal medicine (52). In a subsequent report conducted in 2022, stakeholders concluded the need to develop a long-term strategy to grow the Canadian veterinary population a net annual rate of 3.5%–4% over the next 10 years (53). Food animal veterinarians play an important role in animal health and welfare, the management and prevention of diseases, public health, biosecurity and food quality (8–10, 12). Therefore, initiatives to combat this perceived and projected shortage of food animal veterinarians are important to both human and animal health.

Several solutions to the shortage of food animal veterinarians entering practice have been proposed such as: modifications to the selection process for veterinary schools, debt repayment, increased mentoring opportunities for food animal medicine students, monetary incentives and increased food animal focused learning early in the curriculum (10, 23, 54). Another solution to the shortage could be to promote telemedicine veterinary services for livestock producers. Several participants mentioned using telemedicine services to send photos and videos to their veterinarian for diagnosis and treatment plans, suggesting that telemedicine may be a viable option for bison producers. Telemedicine has already been used in the beef feedlot and swine industries for services like post-mortems, and have proven to be a viable alternative for in person visits (55, 56). Telemedicine can also be used as a teaching tool to increase knowledge and help build new technical skills among producers (57). However, there may be limitations to utilizing telemedicine to teach veterinary technical skills for producers with small herd sizes, as it could be challenging for producers to become comfortable enough to use technical skills on their own. This may not be a limitation for our sample of Ontario bison producers as the results of our study indicate they often handle and restrain their herds for treatments without any additional help. Similar to the steps taken by the United States Department of Agriculture, providing monetary support for telemedicine development and distance learning may also increase implementation across food animal practices (58).

Participants identified the lack of perceived bison-related knowledge among veterinarians as their most prominent barrier to accessing veterinary services. As a result, most producers chose to contact other producers to solve herd health concerns and identify ailments before calling a veterinarian. This choice could have potential impacts on delayed treatment or reporting of herd health issues. For example, a 2019 Australian study reported that not seeking veterinary services may result in the underreporting of diseases in surveillance systems (55). It is important to note that, despite participants choosing to contact other producers before contacting a veterinarian, they felt they were still able to provide their herds with appropriate care. Studies have shown some common information sources for producers in the beef and dairy industries include veterinarians, peer networks, other producers, government agencies, industry personnel (e.g., feed and farm supply stores), producer organizations, the internet and university specialists (59–62). Another study found that producer satisfaction with their veterinary professional was positively correlated with their willingness to adopt recommendations (63). This suggests that if producers are dissatisfied with the advice or service their veterinarian provides, or if they lack trust in their veterinarian's ability to treat their animals effectively, they may be more inclined to seek guidance from other producers who they trust and have a more positive relationship with.

Given that veterinary involvement on farms can result in improved herd health and welfare, higher productivity, and early problem detection, facilitating relationships between veterinarians and bison producers may lead to improvements in health and welfare among bison herds (18, 19). In companion animal research, establishing a strong veterinarian-client-patient relationship positively influences client adherence to veterinary recommendations and treatment plans, ensuring animal health (64). Strategies to help develop stronger relationships between veterinarians and bison producers and bridge the gap in bison-related knowledge include both hands-on experience and theoretical learning opportunities. Proposed strategies include incorporating farm visits for both established veterinarians and veterinary students, in addition to providing veterinary students with resources on niche species. Farm visits could be organized by the Ontario Bison Association with participating farms and would provide veterinarians with the opportunity to complete Continuing Professional Development hours as recommended by the College of Veterinarians of Ontario (65).

While participants indicated they all had access to large animal veterinarians—even if they chose not to use them—many of the available veterinarians were not familiar with bison. This differs from a previous report indicating food animal producers had limited access to veterinary services in rural Ontario, which may be a result of lack of representation of bison producers in the study group (11). A US study conducted in 2013 found that just over 44% of sampled small-scale livestock producers including beef, dairy, swine, sheep, goat and poultry, did not consult a veterinarian for medical concerns due to the veterinarian being too far away or not having access to one (30). A study that surveyed Tennessee livestock producers, including dairy, beef, poultry, swine and horses, found that 18.8% were unable to find specialized veterinary services to treat their needs (23). Having fewer veterinarians with species-specific knowledge may increase their caseloads which, in a 2022 study, was identified as a key indicator for burnout (66). Experiencing burnout can impact veterinarians' communication with producers, inadvertently pushing producers to seek advice from more readily available sources like other producers (67). Improving bison-specific knowledge among large animal veterinarians could decrease the call-out time for a veterinarian with species specific knowledge. Similar strategies focused on strengthening relationships and utilizing both theoretical and hands-on learning opportunities to bridge knowledge gaps should be employed. These strategies could include providing credible physical resources about bison to veterinarians and hosting farm visits (outside of medical calls) for both graduated veterinarians and veterinary students. These strategies could help increase bison-specific knowledge among incoming and experienced veterinary practitioners.

### 4.1 Limitations

As our study used a qualitative approach, the findings are not meant to represent the entire population of bison producers but rather represent the experiences, thoughts, opinions, and perceptions of those who participated. The nature of qualitative research is inherently molded by the research team, as they conceptualize, interpret, and report the findings of the study (68). Our team's interpretation of the data was influenced by our qualitative research experience and our backgrounds in epidemiology and veterinary sciences. Our unfamiliarity with both bison farming and bison producer perspectives may have influenced our analysis. We used convenience sampling to obtain our participants, which may not have achieved maximum diversity in perspectives as other sampling techniques (e.g., purposive sampling). However, our final sample represented 20% of the population and participants were varied in aspects like age, industry experience, herd size, time in industry, management practice style and geographic location. Furthermore, it is important to note that this study focused on producers' perspectives on their access to veterinary services; therefore, we did not capture the views of veterinarians. Further research is needed to explore veterinarians' perceptions and experiences with treating bison herds in Ontario.

## 5 Conclusion

We aimed to describe bison producers' access to veterinary services in Ontario, Canada and to identify how any perceived barriers might affect producers' herd health strategies. The findings suggest that producers have concerns surrounding the number of new, large animal veterinarians entering the industry, increasing costs associated with veterinary services, and longer call-out times for veterinarians with bison-knowledge. One of the most prominent findings from the study was the producers' desire to improve bison-specific knowledge among veterinarians servicing their farms. The findings from this study can be used to better inform policies and curriculum development, develop support networks, and act as a starting point for additional research into unique food producers' access to veterinary services. Further research into the veterinarian perspective would add additional value and contribute to a more complete view of the problem and better inform potential solutions.

## Data Availability

The datasets presented in this article are not readily available because the dataset cannot be made publicly available due to confidentiality reasons. Requests to access the datasets should be directed to Kelsey L. Spence, kspenc04@uoguelph.ca.
