# Supplementary material for: Exploring bison producers' access to veterinary services in Ontario, Canada
Source: Front Vet Sci. 2024 Dec 11;11:1448216. doi: 10.3389/fvets.2024.1448216 (PMC11668789; doi:10.3389/fvets.2024.1448216)
Supplement: Supplementary file 1 [file Data_Sheet_1.pdf]

Supplementary Material

1 Supplementary Data

1.1 Supplementary Data

<u>Focus Group Guide</u>

Introduction/Welcome: Welcome everyone, thank you for taking the time to participate in this focus group to support my research project. My name is Natassia Lambrou and I'm a MSc student under the supervision of Drs. Kelsey Spence & Charlotte Winder at the OVC. As part of my degree, I am conducting an exploratory study looking at your experiences and access to veterinary care in Ontario. Our research study aims to understand how we can help ensure you have healthy farms and where there are, if any, gaps to your access to veterinary services for your bison. Your participation in this study will help us describe your current level of access and will help us determine if there's anything we can improve on to help you with your bison farms. The results from this study could have the potential to help develop new guidelines within governing bodies. I'll ask you some prompting questions during this session, but the idea is to have an organic free-flowing discussion on your thoughts surrounding your access to veterinary care for your herd.

Consent/How the group will run: The focus group will run from 60-90minutes in length and will be recorded for transcription purposes. We will be on a first name basis today. With your permission, anonymized statements from the discussion may be used in publications and/or presentations. Given the group format of this session I will ask you to keep in confidence information that identifies or could potentially identify a participant and/or their comments. I will be acting as the moderator for the session and will encourage everyone to participate. There are no right or wrong answers to the questions, just different opinions – and everyone's opinions are valid. Please share your point of view even if it differs from others in the group. You may decline to answer any question(s) during the discussion. You may decide to leave the study at any time by communicating this to me. We'll operate in a mute/unmute fashion for today's session, so if you have a question or a point to bring up, please feel free to unmute to answer and discuss. If we're having a discussion, please feel free to keep your mics unmuted so you can engage more readily. As for cameras, that is personal preference, I will have mine on throughout the discussion.

- To get to know you all better I would like to ask a simple question to start: how long have you all been farming bison for?
    - What initially made you want to pursue bison farming?
- What is a typical day like for you on the farm?
    - Who helps you?
    - What are your typical duties in a day?
    - Who might you call if you needed a hand on the farm?
    - Would this change based on the situation?
- Scenario: You arrive on farm in the morning and see a couple of your calves are scouring and looking dull – who would you call?
    - Veterinarian: how long would it take for the vet to be able to see you? Would you do a phone consultation? If so, why?
    - Fellow Producer: why would you decide to call a producer instead of a veterinarian?
    - Wouldn't call anyone: you say you wouldn't call anyone, why? And what are your next steps?
- Do you work with a veterinarian regularly?
    - Yes:
    - How did you choose your veterinarian?
    - Tell me more about your relationship and experience with your vet
        - What is the best part of your relationship with your veterinarian? Tell me more
        - Is there anything that could be improved in your relationship with your vet? Tell me more
            - How often do you call your vet, might even answer this with the above questions
            - Does anyone else have a similar experience to so and so?
            - What do you think of your vet's availability to visit your farm?
            - What are some other veterinary services that aren't current available to you that you think would be beneficial to you and your farm?
            -
    - No:
        - How do you manage herd health on the farm? Tell me more about that.
        - How might seeing a veterinarian change how you currently manage herd health?
        - Would anyone else take a different approach? Tell me about it.
    - Are there any obvious barriers to access to care that you as a bison producer experience?
        - Tell me about them

        - If there was one barrier that you could remove to have the biggest positive impact on herd health, which one would it be?
- When was the last time you contacted a vet?
    - Tell me about the circumstances led you to contact a vet?
        - Would you/have you called a vet for nutrition concerns
        - Would you/have you called a vet for parasite prevention planning
        - Would you/have you called a vet for breeding procedures/concerns
    - Walk me through that experience
        - Positive: what aspects of the visit made it positive? Knowledge, timeliness, ability to handle/care for the bison?
        - Negative: what aspects of the visit made it negative? Knowledge, timeliness, ability to handle/care for the bison?
        - Neutral: did any aspects of the visit stand out to you? Knowledge, timeliness, ability to handle/care for the bison?
- Last question I have for you all is, what is one thing that is going really well from a herd management perspective on your farm?

<u>Conclusion:</u> This has been a wonderful discussion; a lot of interesting points were brought up. I will end our focus group here, thank you again for your participation. Please monitor your email for the anonymized results if you indicated you wanted to receive them in the entrance survey. Have a lovely rest of your day, thank you again.
